# The Rac sign: a transthoracic echocardiography sign that should prompt thorough evaluation of the coronary arteries

**DOI:** 10.1093/ehjcr/ytaf300

**Published:** 2025-07-10

**Authors:** Panagioula Niarchou, George Michas, Efstathia Prappa, Athanasios Trikas

**Affiliations:** Department of Cardiology, Evangelismos General Hospital of Athens, 45-47 Ipsilantou St, 106 76 Athens, Greece; Department of Cardiology, Evangelismos General Hospital of Athens, 45-47 Ipsilantou St, 106 76 Athens, Greece; Department of Cardiology, Evangelismos General Hospital of Athens, 45-47 Ipsilantou St, 106 76 Athens, Greece; Department of Cardiology, Evangelismos General Hospital of Athens, 45-47 Ipsilantou St, 106 76 Athens, Greece

## Case description

**Figure 1:**
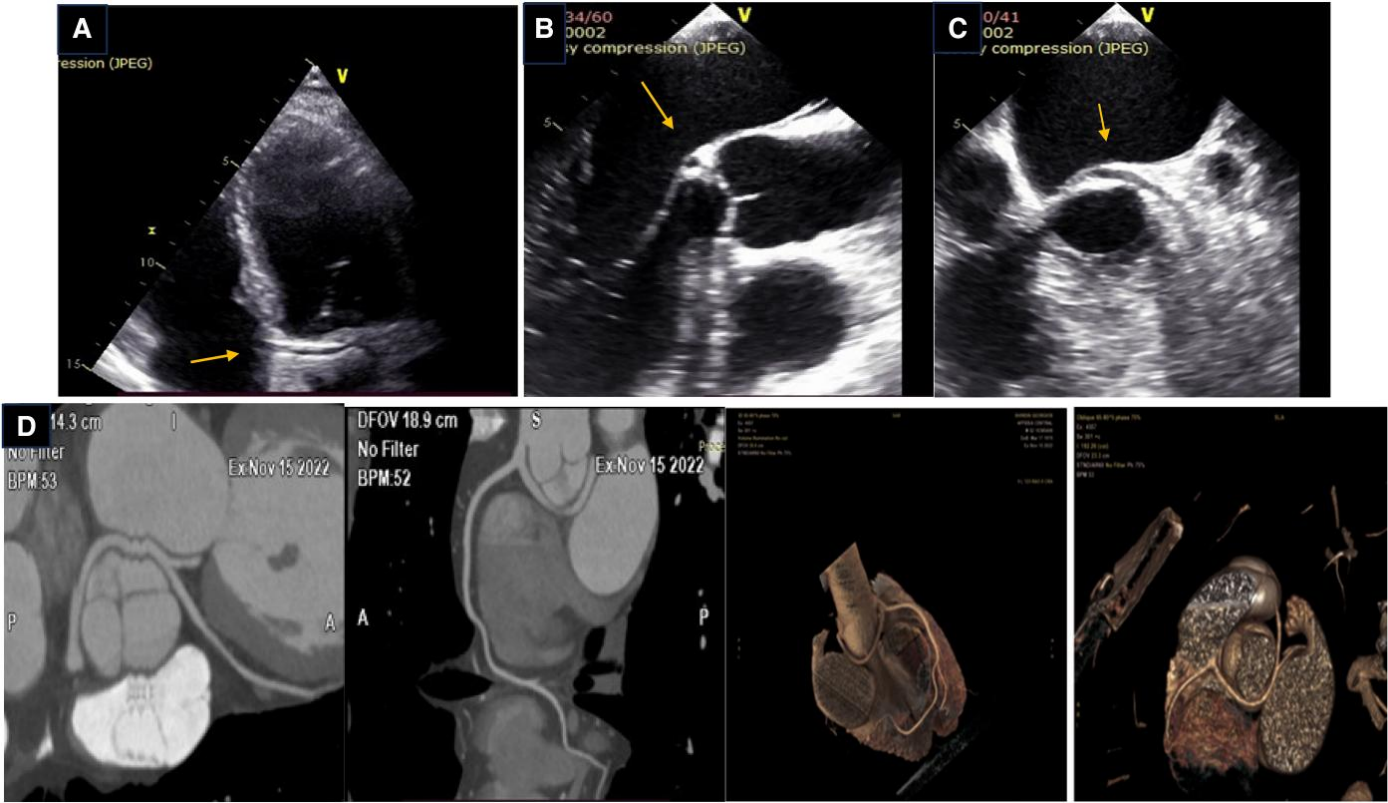
(*A*) Transthoracic echocardiography. Apical 5-chamber view. ‘RAC’ sign—a linear structure with vascular characteristics crossing transversely to the aorta (arrow). (*B*) Transoesophageal echocardiography. Mid-esophageal view at 120°: a circular structure below the non-coronary cusp, corresponding to the ‘bleb’ sign. (*C*) Transoesophageal echocardiography. Mid-esophageal view at 50°; the aorta is shown in short axis. A vascular structure with a retroaortic course is observed (arrow). (*D*) Coronary Computed Tomography Angiography. 2D slices and 3D reconstruction of the retroaortic course of the left main (LM) and the left coronary artery originating from the proximal right coronary artery.

A 56-year-old man was referred for routine checkup. He had medical history of arterial hypertension and engaged in intense sports activity. He was asymptomatic, with no history of syncope or angina. His ECG showed slight left axis deviation and sinus bradycardia. Transthoracic echocardiography revealed normal left ventricular volumes and global systolic function. Apical 5-chamber view revealed a vascular structure in the atrioventricular groove above the mitral annulus plane, crossing the aorta perpendicularly—consistent with the ‘retroaortic course’ (RAC) sign (*Figure 1A*; see Supplementary material online, *Video S1*).^1^ Detailed evaluation of the coronary artery (CA) origin in the short-axis view illustrated right CA origin, but left main origin failed to be identified. Moderate mitral regurgitation of unclear aetiology was also noted, prompting a transoesophageal study (TEE), before patient referral for Coronary Computed Tomography Angiography (CCTA). TEE confirmed the ‘RAC’ sign, and, in the long-axis view of the aorta below the non-coronary cusp, revealed a circular structure likely corresponding to the ‘bleb’ sign.^2^ (*Figure 1B,C*; see Supplementary material online, *Video S2*). Mitral valve appeared structurally normal. CCTA that followed revealed an anomalous origin of the left main from the proximal part of the right CA with a subsequent retroaortic course (*Figure 1D*; see Supplementary material online, *Video S3*), an extremely rare and potentially malignant anatomy since all three coronary branches originated from the RCA. Following ESC guidelines, the patient underwent a nuclear stress test on stationary bicycle, with no signs of ischaemia. Since there were no other high-risk features on CCTA, the patient was managed conservatively with strict cardiovascular risk control.

Coronary artery anomalies (CAAs) refer to a group of congenital conditions including either an abnormal origin or course of any of the three main epicardial coronary arteries. While CCTA remains the gold standard for the diagnosis,^3^ routine echocardiography, particularly the RAC sign, can raise suspicion of a CAA, with reported high specificity (93.9%) compared with other echocardiographic markers.^1^

## Supplementary Material

ytaf300_Supplementary_Data

## Data Availability

The data underlying this article will be shared upon reasonable request to the corresponding author.

## Notes

**Consent:** Written informed consent for submission and publication of this case report including images has been obtained from the patient in line with COPE guidance.

**Funding:** None declared.
